# Subacute and Reproductive Oral Toxicity Assessment of the Hydroethanolic Extract of *Jacaranda decurrens* Roots in Adult Male Rats

**DOI:** 10.1155/2013/414821

**Published:** 2013-11-17

**Authors:** Joyce Alencar Santos, Aline Arruda, Claudia Andrea Lima Cardoso, Maria do Carmo Vieira, Ana Cláudia Piccinelli, Diana Figueiredo de Santana Aquino, Cândida Aparecida Leite Kassuya, Arielle Cristina Arena

**Affiliations:** ^1^School of Health Sciences, Federal University of Grande Dourados, Caixa Postal-322, Dourados, MS, Brazil; ^2^Mato Grosso do Sul State University, Caixa Postal-351, Dourados, MS, Brazil; ^3^School of Agrarian Sciences, Federal University of Grande Dourados, Caixa Postal-533, Dourados, MS, Brazil; ^4^Department of Morphology, Institute of Biosciences of Botucatu, São Paulo State University (UNESP), Distrito de Rubião Junior, S/N, Caixa Postal-510, 18618970 Botucatu, SP, Brazil

## Abstract

*Jacaranda decurrens* subsp. *symmetrifoliolata* Farias & Proença (Bignoniaceae) is a species traditionally used for the treatment of inflammatory and infectious diseases. Previous findings from our group reported scientifically that *J. decurrens* has anti-inflammatory efficacy. However, more toxicological studies are needed to support and ensure its safe use. The present study was carried out to evaluate the toxic effects of a prolonged treatment with hydroethanolic root extract of *J. decurrens* (EJD) on hematological, biochemical, and reproductive parameters in adult male rats. The animals received by oral gavage 0; 250; 500; or 1000 mg/kg body weight of EJD for 28 days. After the treatment, biochemical, hematological, histopathological, and reproductive parameters were analyzed. The EJD treatment did not cause adverse effects on body weight gain, feed and water consumption, hematological and biochemical profiles, or histopathological analysis of liver and kidney. Similarly, there were no statistically significant differences in reproductive parameters, such as sperm production, number of sperm in the epididymis, and sperm morphology. These results demonstrate the absence of subacute toxicity as a result of the oral treatment with EJD for 28 days in adult male rats. However, other studies should be performed to evaluate the total safety of this plant.

## 1. Introduction 

In recent years, natural health products have been increasingly used worldwide, particularly herbal products [1]. One reason for the widespread use of medicinal species is the belief that these products from medicinal plants are risk free and considered by patients to be a safe alternative for the treatment of several diseases [2]. However, few plants selected for medicinal use have been scientifically studied to assess their quality, safety, and efficacy [3]. Thus, studies of acute toxicity and toxicity after repeated doses are required by the Brazilian Health Surveillance Agency (ANVISA) to regulate and register phytotherapic products in order to evaluate the safety of their use [4].

*Jacaranda decurrens* subsp. *symmetrifoliolata* Farias & Proença (Bignoniaceae), traditionally known as “carobinha-do-campo,” “carobinha,” or “caroba,” is an endemic species found in the Brazilian states of Goias, Mato Grosso, Mato Grosso do Sul, Minas Gerais, and São Paulo [5]. According to folk medicine, the leaves and/or the roots were prepared in form of infusion, decoction, and “garrafadas” against inflammatory diseases and infections [6]. 

Pharmacological evaluations have reported that species from *Jacaranda* genus have antioxidants [7], antimicrobials [8], and chemopreventive properties [9]. Recent studies performed by our group showed that *J. decurrens* extract has anti-inflammatory properties in adult rats [10]. 

Although studies have demonstrated their therapeutic effects, few toxicological studies conducted on *J. decurrens* can be found in literature. After performing acute toxicity tests, no signs of toxicity were observed at doses of 2000 or 5000 mg/kg of leaves [8] or 500, 1000, or 2000 mg/kg of roots of *J. decurrens* extract [10] in mouse or rats, respectively. However, the aqueous extract of *J. decurrens* roots administered to pregnant rats interfered with the onset of reproductive development in male offspring, anticipating the age of testicular descent and reducing absolute and relative weight of testis and epididymis of the animals at 60 days of age [11], which reinforces the need to evaluate the safety of its use through different experimental protocols standardized by regulatory agencies.

Due to the scarce information in the literature about the toxicological profile after subacute exposure and evidence of reproductive disturbances in male offspring after maternal exposure to *J. decurrens* extract, the aim of this study is to evaluate the toxic effects of hydroethanolic root extract of *J. decurrens* in adult male rats through hematological, biochemical, and reproductive parameters.

## 2. Materials and Methods 

### 2.1. Plant Material, Preparation, and Isolation of Extract

*J. decurrens* subsp. *symmetrifoliolata *roots (EJD) were collected (April 2010) in the Medicinal Plant Garden of the Federal University of Grande Dourados. A voucher specimen was identified by Dr. Rosana Farias Singer and deposited (register: W. G. Garcia 14.008) in the Herbarium of the Department of Botany at the Biology Institute of the State University of Campinas. 

The roots of *J. decurrens* were dried at a temperature of ±30°C. The dry roots (560 g) of *J. decurrens* were extracted with 3.5 L of ethanol : water (70 : 30 v : v) at room temperature. Each extraction was performed with 700 mL by seven days, amounting to 3500 mL. The procedure was performed in quintuplicate. Extracts were united, filtered, and concentrated under vacuum and lyophilizer. During the treatment, the extract was dissolved in a hydroethanolic solution. 

The EJD (1.14 g) was dissolved in water (0.2 L) and fractionated by XAD-2 (Supelco, Bellefonte, PA, USA) resin column chromatography (30 × 3 cm) eluted with water (0.3 L), followed by methanol (0.2 L) and with ethyl acetate (0.2 L). The water and ethyl acetate fractions were concentrated in employing nitrogen. The fractions obtained were dissolved with 5000 *μ*L of methanol, filtered through a membrane with pores of 0.22 *μ*m, and analyzed using high performance liquid chromatography (HPLC). The compounds were employed as standards quercetin, rutin, myricetin, myricetrin, luteolin, and quercetrin. The fractions were analyzed in an analytical LC (Varian 210) system with a ternary solvent delivery system equipped with an autosampler, a photodiode array detector (PDA) monitored at *λ* = 200–800 nm. The LC column was a C-18 (25 cm × 4.6 mm; particle size, 5 *μ*m; Luna, Phenomenex, Torrance, CA, USA), with a small precolumn (2.5 cm × 3 mm) containing the same packing, used to protect the analytical column. In each analysis the flow rate and the injected volume were 1.0 mL/min and 20 *μ*L, respectively. All chromatographic analyses were performed at 25°C. Elution was carried out using the following solvent-gradient program : water : methanol (70 : 30, v/v) by 25 min, taking 15 min to reach 100% methanol, then returning in exactly 5 min to the initial conditions. The identification was made by comparison of retention times and UV spectra of peaks of the samples with the peak standards.

### 2.2. Animals

Adult male Wistar rats (90 days old, weighing approximately 340 g, *n* = 32) from the Federal University of Mato Grosso do Sul were maintained under controlled temperature (23°C), with a constant 12 h light-dark cycle and free access to food and water. The experimental procedures were in accordance with the Ethical Principles in Animal Research and approved by the Committee for Ethics in Animal Experimentation at the University Center of Grande Dourados (Protocol number: 334/10). 

### 2.3. Doses and Treatment

The toxicity studies were based on the Organization for Economic Cooperation and Development (OECD)—Guideline 407 and ANVISA guidelines (Brazilian Health Surveillance Agency) [12, 13]. Adult male rats were divided into four groups. The treatments were performed by a single oral (gavage) administration at doses of 0; 250; 500; or 1000 mg/kg of body weight of EJD for 28 consecutive days. The control group received only vehicle (hydroethanolic solution). 

### 2.4. Evaluation of Subacute Toxicity

The animals were weighted daily and observed for clinical signs of systemic toxicity. Behavior parameters, death, their weight, and the amount of water and feed were analyzed [12]. 

After treatment, the experimental group animals were weighed and anesthetized (ketamine and xylazine, 25 and 10 mg/kg, resp.). Next, blood samples were collected from the renal vein, with and without anticoagulant (Heparin sodium, Cristália). The blood samples were used to determine the hematology parameters (total and differential leukocyte count, hematocrit, hemoglobin, and erythrocyte count), and the nonanticoagulated serum samples were used for biochemical analysis (aspartate aminotransferase—AST, alanine aminotransferase—ALT, gamma-glutamyl transferase—*δ*-GT, creatinine, and urea) [12, 14]. The semiautomatic Bioplus Bio200 equipment (Gold Analysis kits) was used to determine the biochemical parameters. After that, the animals were euthanized, the vital organs (liver, lung, and right kidney) were removed, and the weight (absolute and relative to body weight) was determined. For the histopathological evaluation of these organs, the samples were fixed in 10% buffered formalin and processed for histological study by light microscopy. The parameters investigated were reversible (degeneration) and irreversible cell damage (necrosis and apoptosis), leukocyte infiltration, congestion, extravasation of blood, and fibrosis. 

### 2.5. Evaluation of Reproductive Toxicity

The reproductive organs (testis, epididymis, ventral prostate, vas deferens, and seminal vesicle) of the experimental group animals were removed and weighed (absolute and relative to body weight). For standardization, the right testis and epididymis were used for the germ cell counts, and the left organs were fixed for histopathological evaluation, using the same method described earlier for the vital organs. 

### 2.6. Daily Sperm Production per Testis, Sperm Counts, and Transit Time in the Epididymis

Homogenization-resistant testicular spermatids (stage 19 of spermiogenesis) and sperm in the caput/corpus and cauda epididymis were enumerated as described previously by Robb et al. [15], with adaptations adopted by Fernandes et al. [16]. Briefly, each right testis, decapsulated and weighed soon after collection, was homogenized in 5 mL of NaCl 0.9% containing Triton X 100 0.5%, followed by sonication for 30 s. After a 10-fold dilution, a sample was transferred to Neubauer chambers (4 fields per animal), preceding a count of mature spermatids. To calculate daily sperm production (DSP), the number of spermatids at stage 19 was divided by 6.1, which is the number of days of the seminiferous cycle in which these spermatids are present in the seminiferous epithelium. In the same manner, caput/corpus and cauda epididymidis portions were cut into small fragments with scissors and homogenized, and sperm counted as described for the testis. The sperm transit time through the epididymis was determined by dividing the number of sperm in each portion by the DSP. 

### 2.7. Sperm Morphology

For evaluation of sperm morphology, the interior of the left vas deferens of mature rats was washed, by the aid of a syringe and needle, with 1.5 mL were prepared of saline solution, after which histological slides. Two hundred spermatozoa (heads only or intact sperm) per animal were evaluated for head and/or flagellar defects by phase-contrast microscopy (×200, total magnification) in wet preparations [17].

### 2.8. Statistical Analysis

For comparison of results among the experimental groups, statistical tests for analysis of variance were utilized—ANOVA, with the “a posteriori” Tukey-Kramer test. Differences were considered significant when *P* < 0.05. The statistical analyses were performed by GraphPad InStat (version 3.02). 

## 3. Results and Discussion

Toxicological evaluations after repeated exposures are required by regulatory agencies to characterize the toxicological profile of any substance [12, 13]. However, there is little toxicological information in the literature to support and ensure its safe use. To our knowledge, the present study represents the first research that demonstrates the absence of subacute toxicity of extract of *J. decurrens* roots in adult male rats. 

Mortality is a clear sign of toxicity; however, other variables may be indicative of more subtle adverse effects, such as loss of body mass during the treatment and clinical signs of toxicity (diarrhea, piloerection, and changes in behavior) [12]. Male rats exposed to the extract of *J. decurrens* roots did not exhibit clinical signals of toxicity at the doses tested. The subacute oral toxicity study also showed no significant changes in the amount of water and feed between the experimental groups (data not shown). Additionally, the absolute and relative weight of the vital organs (liver, kidney, and lung) (Table 1) showed no statistically significant differences in any of the doses tested. 

The hematopoietic system is one of the targets more susceptible to toxic substances and is an important parameter for assessing the physiological and pathological status in humans and animals [18]. After the hematological analysis, it was observed that the values of hematocrit, hemoglobin, platelet count, and erythrocyte count, total and differential leukocyte of the treated animals were similar to controls (Table 2), indicating that the extract provided no adverse effects on blood cells circulating or on their production. 

Clinical biochemistry determinations to investigate major toxic effects in tissues and, specifically, effects on kidney and liver should be performed and under certain circumstances, they may provide useful information. Some enzymes can be used as indicative of hepatocellular effects (such as ALT, AST, and *δ*-GT) [12, 19] and others as biomarkers of nephron functional injury (creatinine, urea) [20]. The biochemical analysis (AST, ALT, *δ*-GT, creatinine, and urea) in the present study did not differ significantly in the experimental groups (Table 2). Similarly, there were no significant changes in relation to the absolute and relative weight of vital organs (liver, lung, and kidney) (Table 1) or regarding the histology of these organs (data not shown). Thus, in the same way as after an acute exposure [10], the exposure to multiple doses of *J. decurrens* roots also did not cause systemic toxicity. Therefore, it is not possible to generalize that the species *Jacaranda decurrens* has no toxicity, and more studies are necessary to conclude this fact.

Reproductive toxicity studies must be regarded as part of an herbal safety evaluation process which supplements toxicological systemic tests [21]. Therefore, this study evaluated some well-validated reproductive parameters in reproductive toxicology. One of the protocols of the Organization for Economic Cooperation and Development (OECD) recommends evaluating the reproductive/developmental toxicity associated with the administration of repeated doses. This protocol can be used to provide initial information on possible effects on male and female reproductive performance such as gonadal function, mating behaviour, conception, development of the conceptus, and parturition, either at an early stage of assessing the toxicological properties of chemicals or on chemicals of concern [22]. 

In this study, no significant differences were observed in absolute and relative weight of the most reproductive organs (testis, epididymis vas deferens, and seminal vesicle) of animals exposed to EJD (Table 1). However, the weight of the prostate was higher in animals exposed to 250 and 500 mg/kg of extract, in relation to control group. As variations in reproductive organ weights can be used as a parameter for indicating changes in the sex hormones levels, [23] suggests that subacute treatment with EJD may have not changed the hormonal status (androgens) in the animals. However, further studies, such as hormonal analysis, are needed to confirm this hypothesis. 

**Figure 1:**
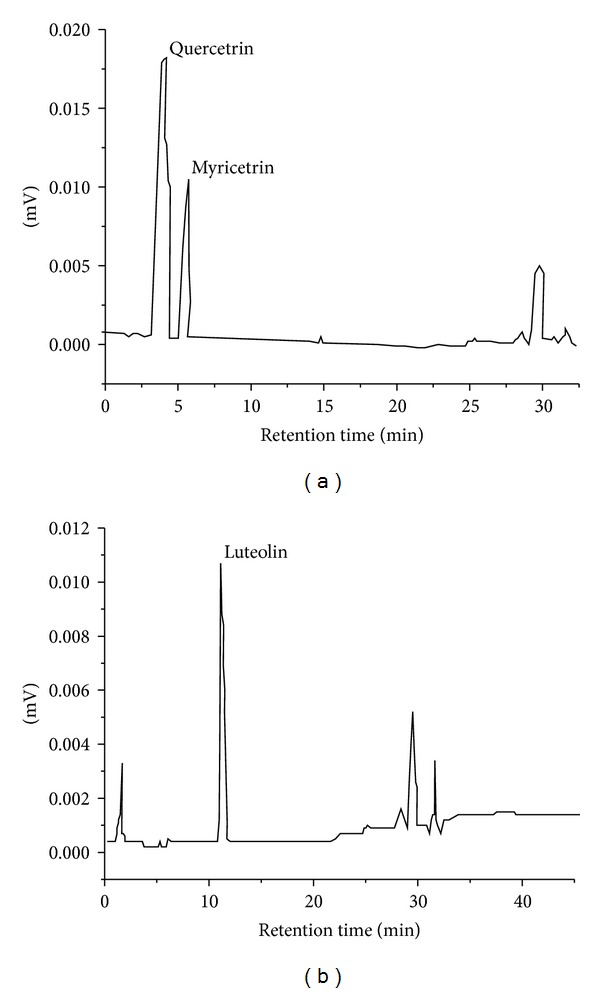
Chromatogram of myricetrin and quercetrin in water fraction in the *λ* = 340 nm (a), and luteolin in ethyl acetate fraction in the *λ* = 350 nm (b) of the *Jacaranda decurrens* extract.

Previous findings from our group indicated that the oleanolic and ursolic acids (triterpenoids) were isolated in the methanolic fraction of the EJD roots [10]. In other study, analyses performed using HPLC indicated the presence of ursolic acid in fractions of the *J. decurrens* extract [7]. In the present study, the presence of flavonoids luteolin in ethyl acetate fraction and myricetrin and quercetrin in water fraction of the extract was identified (Figure 1). 

Flavonoids and triterpenoids are substances naturally found in a large variety of vegetarian foods and medicinal plants. Despite its beneficial effects on health and its relatively low toxicity [24], some isolated EJD components have been reported to have adverse effects on reproduction. Oleanolic acid, extracted from *Eugenia jambolana*, was shown to have antifertility effects in male rats [25]. Oleanolic acid and other triterpenoids have also been shown to be an inhibitor of testosterone 5*α*-reductase and to have antimale hormone activities [26]. Thus, these compounds can disrupt the endocrine system, affecting sperm production and/or the number of sperm in the epididymis, compromising fertility in the animals exposed [27]. A toxic agent can affect the maturation and function and sperm survival by acting directly on the sperm or changing the epididymal function [28]. 

Yakubu et al. [29] demonstrated that medicinal plants can alter the male reproductive system, by testing the aqueous extract of *Chromolaena odorata*, at doses of 250 and 500 mg/kg, evidencing its antiandrogenic activity, since there was a reduced number of sperm in the male rats exposed orally for 14 days. In the same way, Arena et al. [11] observed a change in the initial development of the male reproductive system, characterized by anticipation of the age of testicular descent of the pups and a decrease in the relative weight of testis and epididymis of animals during the puberty stage, after a gestational and lactational exposure to aqueous extract of *J. decurrens*. Controversially, in the present study, after sperm counts in the testis and epididymis, no changes were observed in the sperm production or in the epididymal sperm number (Table 3). Morphological analysis of sperm extracted from the vas deferens showed that the percentages of normal (control = 86.32%; 250 mg/kg = 85.15%; 500 mg/kg = 87.27%; 1000 mg/kg = 86.75%, *n* = 8) and abnormal forms were similar among the groups. In the same way, the testis and epididymis analysis by light microscopy did not reveal any morphological changes related to the treatment (data not shown). Conflicting results may be due to differences in the experimental protocol of these two studies, such as the treatment period (during pregnancy or adult life) and the vehicle (aqueous or hydroethanolic) used in the extract preparation. 

## 4. Conclusions 

These results demonstrate the absence of subacute toxicity due to oral treatment with EJD for 28 days in adult male rats. Furthermore, in this experimental model, EJD not interfere with the male reproductive system of rats, since the evaluated parameters were not affected. However, other studies based on protocols elaborated by regulatory agencies should be performed (such as studies of chronic toxicity, genotoxicity, analyse of the level of protein, glucose, potassium, and total cholesterol) in order to evaluate the total safety of this plant.

## Figures and Tables

**Table 1:** Body weight and relative organs weights of animals exposed to *Jacaranda decurrens* in the subacute toxicity study.

Parameter	Control (vehicle)	250 mg/kg	500 mg/kg	1000 mg/kg
Body weight (g)	430.00 ± 22.31	422.00 ± 22.10	439.00 ± 32.21	426.00 ± 18.20
Liver (g/100 g)	2.78 ± 0.07	2.74 ± 0.06	2.67 ± 0.21	2.70 ± 0.10
Lung (g/100 g)	0.43 ± 0.16	0.49 ± 0.03	0.44 ± 0.02	0.42 ± 0.02
Kidney (g/100 g)	0.31 ± 0.01	0.32 ± 0.01	0.30 ± 0.01	0.29 ± 0.01
Testis (g/100 g)	0.49 ± 0.02	0.47 ± 0.01	0.44 ± 0.01	0.43 ± 0.01
Epididymis (mg/100 g)	147.00 ± 0.01	167.00 ± 0.01	157.00 ± 0.01	158.00 ± 0.01
Prostate (mg/100 g)	124.00 ± 0.02	155.00 ± 0.02*	166.00 ± 0.01*	122.00 ± 0.01
Vas deferens (g/100 g)	0.04 ± 0.00	0.08 ± 0.04	0.14 ± 0.09	0.03 ± 0.00
Seminal vesicle (g/100 g)	0.43 ± 0.02	0.40 ± 0.02	0.37 ± 0.01	0.40 ± 0.01

Values expressed as mean ± SEM, *n* = 8 animals/group. *P < 0.05 by ANOVA-Tukey Kramer test compared with control group.

**Table 2:** Biochemical and hematological parameters of animals exposed to *Jacaranda decurrens* in the subacute toxicity study.

Parameter	Control (vehicle)	250 mg/kg	500 mg/kg	1000 mg/kg
Creatinine (mg/dL)	1.20 ± 0.15	1.50 ± 0.10	1.20 ± 0.10	1.50 ± 0.10
Urea ( mg/dL)	49.50 ± 3.80	54.60 ± 3.60	53.00 ± 4.50	47.90 ± 3.50
AST (U/L)	114.40 ± 19.40	98.40 ± 13.20	100.20 ± 17.40	104.60 ± 24.40
ALT (U/L)	20.80 ± 2.60	25.20 ± 4.00	22.80 ± 1.70	23.60 ± 3.50
*δ*-GT (U/L)	11.78 ± 0.70	13.30 ± 2.10	12.60 ± 0.80	13.30 ± 2.10
Erythrocyte count (×10^6^) mm^3^	7.50 ± 0.45	6.70 ± 0.50	6.70 ± 2.00	7.30 ± 1.70
Hematocrit (%)	39.50 ± 1.90	42.60 ± 0.60	44.10 ± 1.10	42.50 ± 2.00
Platelet count (×10^3^/*µ*L)	1064.00 ± 48.00	1051.00 ± 20.00	1070.00 ± 27.00	1069.00 ± 32.00
Lymphocytes (%)	69.80 ± 4.30	78.00 ± 1.10	76.30 ± 2.20	80.00 ± 1.64
Eosinophils (%)	1.10 ± 0.10	1.10 ± 0.10	1.10 ± 0.10	1.10 ± 0.10
Monocyte (%)	2.90 ± 0.40	3.20 ± 0.20	2.80 ± 0.40	3.30 ± 0.20
Neutrophils (%)	25.30 ± 4.60	23.80 ± 2.10	22.00 ± 1.00	21.20 ± 0.70

Values expressed as mean ± SEM, *n* = 8 animals/group. *P* > 0.05 by ANOVA.

**Table 3:** Sperm parameters of animals exposed to *Jacaranda decurrens* in the subacute toxicity study.

Parameter	Control (vehicle)	250 mg/kg	500 mg/kg	1000 mg/kg
Daily sperm production (×10^6^/testis/day)	35.02 ± 2.27	35.04 ± 0.95	39.79 ± 0.82	34.89 ± 2.59
Daily sperm production (×10^6^/g/testis/day)	25.25 ± 1.58	23.68 ± 0.72	25.59 ± 0.75	25.80 ± 1.58
Relative sperm count in the caput/corpus epididymis (10^6^/g)	352.23 ± 15.96	339.00 ± 23.52	353.15 ± 17.85	342.00 ± 16.40
Relative sperm count in the cauda epididymis (10^6^/g)	650.79 ± 9.45	642.43 ± 20.32	678.00 ± 19.74	680.00 ± 17.88
Sperm transit time in the caput/corpus epididymis (days)	4.04 ± 0.08	4.14 ± 0.09	3.96 ± 0.15	4.15 ± 0.17
Sperm transit time in the cauda epididymis (days)	4.96 ± 0.27	5.03 ± 0.21	4.59 ± 0.45	5.26 ± 0.22

Values expressed as mean ± SEM, *n* = 8 animals/group. *P* > 0.05 by ANOVA.
